# Understanding the Failure of Medical Therapy in PFO-Associated Stroke and the Benefits of Closure: A Narrative Review

**DOI:** 10.3390/neurolint18010011

**Published:** 2026-01-05

**Authors:** Riwaj Bhagat

**Affiliations:** Department of Neurology, Conemaugh Memorial Medical Center, Johnstown, PA 15905, USA; forriwaj@gmail.com

**Keywords:** patent foramen ovale, PFO, stroke, anticoagulation, paradoxical embolism

## Abstract

Patent foramen ovale (PFO) is present in roughly one quarter of adults and is over-represented among younger patients with cryptogenic ischemic stroke. The past decade has produced compelling evidence from randomized trials showing that PFO closure is beneficial than medical therapy in preventing recurrent ischemic stroke in appropriately selected patients. Despite this, anticoagulation continues to be used when closure is not feasible, declined, contraindicated, or considered after recurrent events. The observation that some patients experience “breakthrough” stroke or transient ischemic attack (TIA) despite therapeutic anticoagulation raises a critical question: why does medical therapy fail in PFO-associated stroke, and why does closure appear superior? This narrative review synthesizes the latest evidence on the pathophysiology of PFO-associated stroke, with attention to mechanisms that remain incompletely addressed by anticoagulation. It analyzes randomized trial data comparing antiplatelet therapy, anticoagulation, and transcatheter closure. It examines the role of high-risk PFO anatomical characteristics, the Risk of Paradoxical Embolism (RoPE) score, and the PFO-Associated Stroke Causal Likelihood (PASCAL) classification in understanding medical therapy failure. Additionally, the review explores whether PFO “type” predicts anticoagulation failure and highlights future research directions needed to further optimize therapy. In conclusion, in appropriately selected patients with high-risk PFO features, closure provides greater stroke risk reduction than medical therapy alone, albeit with small absolute risk differences and a procedural risk of atrial fibrillation.

## 1. Introduction

Patent foramen ovale (PFO) is a remnant of fetal circulation that persists in approximately 25–30% of adults, making it one of the most common congenital cardiac anomalies [1,2,3]. While most individuals remain asymptomatic, PFO is over-represented among patients with cryptogenic ischemic stroke, particularly those younger than 60 years, non-lacunar infarct patterns, and without or few traditional vascular risk factors. Paradoxical embolism via a PFO is estimated to account for ≈5% of all ischemic strokes and up to 10% in younger patients [1].

For many years, management of PFO-associated stroke (PFO-AS) was limited to antithrombotic therapy—either antiplatelet agents or vitamin K antagonists—based largely on observational data. Randomized controlled trials of device closure were initially neutral but were underpowered. More recent, larger, and longer-term trials (RESPECT long-term, CLOSE, REDUCE, and DEFENSE-PFO) have demonstrated that PFO closure, when combined with antiplatelet therapy, significantly reduces recurrent stroke compared with medical therapy alone in selected patients, especially those with high-risk anatomical features [4,5,6,7,8]. However, because overall recurrent stroke rates were low in these trials, the absolute risk reduction was modest (on the order of a few percentage points over several years), and PFO closure was associated with an increased risk of atrial fibrillation in the device group [4,5,6,7,8].

Despite this, anticoagulation remains part of clinical practice. It is used when closure is not available or declined, when there is concomitant deep venous thrombosis (DVT), or when stroke recurs despite antiplatelet therapy. Clinicians increasingly encounter patients with cryptogenic stroke, PFO, and recurrent events despite apparently adequate anticoagulation. Understanding why anticoagulation fails, and why closure appears superior in selected subgroups, is critical for optimizing secondary prevention strategies.

This narrative review pursues four main goals:

1. To summarize the pathophysiology of PFO-AS with emphasis on mechanisms that are incompletely modified by anticoagulation.
2. To review evidence comparing anticoagulation, antiplatelet therapy, and PFO closure for secondary prevention.
3. To discuss how PFO anatomy, Risk of Paradoxical Embolism (RoPE) score, and the PFO-Associated Stroke Causal Likelihood (PASCAL) classification relate to medical therapy failure.
4. To propose future research directions informed by these mechanistic and clinical insights.

## 2. Methods

A literature search (PubMed and Google Scholar) for publications from 2000 to 2025 related to PFO, stroke, and secondary prevention was conducted. Search terms included combinations of “patent foramen ovale,” “cryptogenic stroke,” “PFO closure,” “anticoagulation,” and “antiplatelet”. Evidence from English-language randomized controlled trials, meta-analyses, and high-quality observational studies relevant to PFO and stroke were prioritized. Given the narrative (non-systematic) nature of this review, a formal quality grading of studies was not performed; instead, synthesis of representative data to address the following key objectives was aimed for.

## 3. Pathophysiology of PFO-AS: Why an Anatomic Conduit Matters

### 3.1. Classic Mechanisms

Multiple, potentially overlapping mechanisms have been proposed for PFO-associated stroke:

Paradoxical embolism: The traditional and most widely accepted mechanism in paradoxical embolism-migration of thrombus from the venous system through a right-to-left shunt into the arterial circulation [1,3]. Conditions that transiently increase right atrial pressure (Valsalva maneuvers, coughing, and straining) may open the foramen ovale flap even in the absence of persistent right-to-left pressure gradients [1,3]. Anticoagulation reduces but does not eliminate venous thrombus formation. Residual thrombi, even small ones, may still cross the shunt. Thus, the anatomical conduit remains, providing a pathway for stroke whenever transient pressure reversal occurs.

In situ thrombus formation within the PFO tunnel: Especially in long, aneurysmal, or hypermobile septa where low-flow regions may predispose to in situ thrombus formation, which may be platelet-rich and less responsive to anticoagulation [3].

Associated atrial arrhythmias and atrial cardiopathy: A growing body of evidence suggests that structural and functional atrial abnormalities (dilation, fibrosis, impaired function, or subclinical atrial arrythmias) that predispose to thrombogenesis are independent of, but sometimes co-exist with, the PFO [9].

Coexisting prothrombotic states: Patients with inherited or acquired thrombophilias, malignancy, hormonal therapy, or immobilization may continue forming venous thrombi despite therapeutic anticoagulation. The PFO provides an ongoing anatomical risk [1,9].

The central concept is that the PFO serves as a conduit enabling venous thrombi (or thrombi formed in the PFO itself) to bypass the pulmonary capillary filter and embolize to the brain. Transient elevations in right atrial pressure—during Valsalva maneuvers, coughing, defecation, heavy lifting, or even normal activities—can briefly reverse the interatrial pressure gradient and open the flap valve, permitting paradoxical embolization [1,3].

### 3.2. High-Risk Anatomical Features

Not all PFOs are equal. High-risk features associated with higher odds of stroke or higher recurrence on medical therapy include the following:

- Large right-to-left shunt (often defined as >20 bubbles in the left atrium on contrast echocardiography) [10,11].
- Atrial septal aneurysm (ASA) or marked septal hypermobility (≥10 mm excursion) [7,10].
- Long PFO tunnel and “low-angle” PFO, in which the angle between the PFO tunnel and the inferior vena cava or inferior atrial wall is ≤10°, potentially channeling venous flow toward the left atrium [3,12].
- Prominent Eustachian valve or Chiari network, embryologic remnants that direct venous inflow from the inferior vena cava to the fossa ovalis and PFO, increasing the likelihood that venous thrombi reach the left atrium [13,14,15,16].

These features have been repeatedly linked to higher probability that the PFO is causal and to greater benefit of closure compared with medical therapy including anticoagulation.

### 3.3. Emerging Concept

Recent reviews propose that PFO-AS should be viewed as a multifactorial syndrome rather than a single-mechanism disease. In addition to paradoxical embolism and in situ thrombosis, atrial cardiopathy—structural and electrophysiological remodeling of the atria without overt atrial fibrillation—may coexist and contribute to thrombogenesis [3,9].

## 4. Why Anticoagulation May Fail in PFO-AS?

At first glance, anticoagulation should be an attractive therapy: if venous thromboembolism is the main driver of paradoxical embolism, then preventing its formation (or reducing its size) should prevent stroke. Yet multiple lines of evidence suggest that anticoagulation is not reliably superior to antiplatelet therapy in selected patients with PFO-AS, and it clearly does not match the effect size of PFO closure in high-risk populations [17,18,19,20].

Mechanistically, several factors underlie this apparent failure:

### 4.1. The Anatomic Pathway Remains Intact

Anticoagulation modifies thrombogenesis but does not remove the anatomical shunt. Even with reduced thrombus formation, any venous thrombi that do form still have direct access to the systemic circulation through the PFO when transient right-to-left gradients occur. Closure, in contrast, eliminates the conduit altogether.

This distinction is especially relevant when thrombi arise under circumstances where anticoagulation is less effective—e.g., severe stasis, very large clot burden, or underlying hypercoagulability that overwhelms therapeutic anticoagulation [1,21].

### 4.2. Heterogeneity of Thrombus Biology

Venous thrombi are classically fibrin-rich and responsive to anticoagulants. However, thrombi forming in low-flow pockets (e.g., within a PFO tunnel or regions of atrial cardiopathy) may have mixed platelet–fibrin composition. In these settings, antiplatelet-mediated pathways and local endothelial activation can contribute, and anticoagulation alone may be less effective [3,9].

Moreover, coexistent prothrombotic conditions—cancer-associated thrombosis, antiphospholipid antibodies, and myeloproliferative disorders—may produce recurrent thrombosis despite therapeutic anticoagulation. When a PFO is present, each new thrombus represents another opportunity for paradoxical embolism.

### 4.3. Atrial Cardiopathy and Non-PFO Mechanisms

In some patients initially labeled as having PFO-AS, subsequent monitoring reveals atrial fibrillation, atrial flutter, or biomarkers and imaging consistent with atrial cardiopathy. In such patients, anticoagulation is indeed appropriate, but the recurrent stroke risk may arise from atrial-source thromboembolism, not paradoxical embolism.

The NAVIGATE ESUS trial and related studies highlight that DOACs did not reduce recurrent stroke compared with aspirin in patients with embolic stroke of undetermined source (ESUS), including those with PFO, although some subgroups with left atrial enlargement appeared to benefit [18,20,22]. These findings underscore the complexity of stroke mechanisms in patients with PFO and suggest that atrial cardiopathy must be recognized and treated separately from the PFO conduit.

### 4.4. Real-World Limitations of Anticoagulation

In clinical practice, anticoagulation is vulnerable to:

- Non-adherence and missed doses (especially problematic for DOACs with short half-lives).
- Drug–drug interactions and variable absorption.
- Renal or hepatic impairment altering drug levels.

Even “breakthrough” stroke on anticoagulation may be partly explained by unrecognized periods of subtherapeutic drug exposure. Closure avoids this vulnerability by providing an anatomic solution independent of patient adherence.

### 4.5. Residual Risk Demonstrated in Clinical Trials

The CLOSE trial, which directly compared PFO closure, antiplatelet therapy, and anticoagulation, provides a key data point. Over a ~5-year follow-up, 3 out of 187 patients (1.6%) on anticoagulation had recurrent stroke versus 7 out of 174 (4.0%) on antiplatelet therapy, a difference that was not statistically significant. By contrast, 0 out of 238 patients in the closure arm had a recurrent stroke, compared to 14 out of 235 (6.0%) in the antiplatelet arm. While closure was superior to medical therapy overall (driven by antiplatelet-treated patients), it has never been proven superior to anticoagulation in a head-to-head trial [5,19].

Pooled analyses and meta-analyses combining CLOSE with prior trials (e.g., PICSS and NAVIGATE ESUS) suggest that anticoagulation may modestly reduce recurrent stroke compared with antiplatelet therapy in patients with PFO, but the confidence intervals are wide, heterogeneity is substantial, and bleeding risks are higher [17,18,20]. These data strongly suggest that neither antiplatelet therapy nor anticoagulation alone matches the protective effect of closure in those with high-risk PFO anatomy and high PFO-causality scores.

This integrated view helps explain why simply altering coagulation (with anticoagulants) might not fully abrogate risk: thrombus formation may occur in multiple sites (venous system, PFO tunnel, and left atrium), under different hemodynamic and biological conditions (platelet-rich vs. fibrin-rich clot, local stasis vs. systemic hypercoagulability), and in the presence of a persistent anatomical conduit that is unaffected by medical therapy.

## 5. Evidence Comparing Antiplatelet Therapy, Anticoagulation, and PFO Closure

### 5.1. PFO Closure vs. Medical Therapy

The modern PFO closure trials share several features: they enrolled predominantly patients aged 18–60 years with recent cryptogenic stroke, non-lacunar infarct patterns, and a PFO, and they randomized participants to device closure plus antiplatelet therapy versus medical therapy (antiplatelet or anticoagulant) alone.

Key trials include the following:

- **RESPECT (long-term)**—PFO closure with the Amplatzer occluder vs. medical therapy. Extended follow-up (median ≈ 5.9 years) showed a modest but significant reduction in recurrent ischemic stroke with closure (hazard ratio ~ 0.55), particularly in those with large shunts or ASA [4].
- **CLOSE**—Three-arm trial comparing closure, antiplatelet therapy, and anticoagulation. Closure resulted in zero recurrent strokes (0/238) in the closure group, while anticoagulation showed a non-significant trend toward fewer strokes than antiplatelets (three vs. seven strokes in the anticoagulation vs. antiplatelet arms) [5].
- **REDUCE**—Closure with Gore HELEX or CARDIOFORM occluders plus antiplatelet therapy vs. antiplatelet therapy alone. A significant reduction in clinical stroke and new silent brain infarcts on MRI was shown (1.4% vs. 5.4% at ~3 years; hazard ratio 0.23; 95% CI 0.09–0.62) [6].
- **DEFENSE-PFO**—Focused on high-risk PFO (large shunt, ASA, or hypermobile septum). Closure significantly reduced the 2-year stroke rate (0% vs. ~10%) and the composite outcome of stroke, vascular death, or major bleeding (0 vs. 6 events) compared to medical therapy alone [7].

Individual-patient and reconstructed time-to-event meta-analyses of these and earlier trials (CLOSURE I, PC) demonstrate that PFO closure reduces recurrent stroke by approximately 40–60% compared with medical therapy in selected patients. However, because baseline recurrence rates are low, the absolute risk difference is modest, corresponding to an estimated Number Needed to Treat of approximately 11 over several years, based on pooled event rates [8,23] (Table 1).

### 5.2. Anticoagulation vs. Antiplatelet Therapy

Several analyses have focused on whether oral anticoagulants (OACs) outperform antiplatelet therapy in PFO-AS: A pooled observational analysis by Kent et al. of patients from PICSS and other datasets suggested a trend favoring anticoagulation but did not demonstrate a statistically significant difference, in part due to limited power and heterogeneity [17]. The CLOSE trial’s anticoagulation arm (predominantly warfarin) showed fewer strokes than the antiplatelet arm, but sample sizes were small and confidence intervals overlapped [5,19]. NAVIGATE ESUS, which enrolled patients with ESUS (including many with PFO), found that rivaroxaban did not reduce recurrent stroke versus aspirin and increased major bleeding; subgroup analysis in those with PFO also failed to show clear benefit [18,22]. A recent meta-analysis of DOACs vs. aspirin in ESUS (including NAVIGATE ESUS, RE-SPECT ESUS, ARCADIA, and ATTICUS) concluded there was no reduction in recurrent stroke (relative risk~1) or systemic embolism and an increased bleeding risk with DOACs [20].

Collectively, these data support a cautious conclusion: anticoagulation may offer modest protection beyond antiplatelets in some PFO-AS populations, but the advantage is uncertain, and bleeding risk may offset benefit. No evidence suggests that anticoagulation can match the risk reduction achieved by closure in high-risk PFO patients. No RCTs to date have directly compared PFO closure vs. long-term anticoagulation as a primary hypothesis, and therefore any assertion of closure’s superiority over anticoagulation is based on indirect comparisons.

## 6. PFO Anatomy, RoPE Score, and PASCAL Classification: Linking Structure, Causality, and Medical Therapy Failure

### 6.1. RoPE Score

The RoPE score estimates the likelihood that a detected PFO is causally related to a cryptogenic stroke rather than incidental. It incorporates age, cortical stroke location, history of hypertension, diabetes, prior stroke/TIA, and smoking status. Higher RoPE scores (typically in younger patients with few vascular risk factors and cortical infarcts) imply a higher probability that the PFO is pathogenic [1].

However, RoPE does not include PFO anatomical features. Two patients with identical RoPE scores may have very different PFO anatomy and risk of recurrence.

### 6.2. PASCAL Classification

The PASCAL classification, proposed by Kent and colleagues, integrates the RoPE score with high-risk anatomical features (large shunt and ASA) to categorize patients into groups where the PFO is deemed unlikely, possible, or probable as the cause of stroke [1,10].

- Probable PFO-AS—high RoPE score and at least one high-risk PFO feature.
- Possible PFO-AS—intermediate combinations.
- Unlikely PFO-AS—low RoPE score and absence of high-risk anatomical features.

Application of PASCAL classification to trial datasets demonstrates that the absolute benefit of closure is concentrated in the “probable” group, with more modest or uncertain benefit in other categories [1,11].

It is important to note that both the RoPE score and PASCAL classification have not yet been fully validated in prospective studies, so their use in individual patient decision-making requires clinical judgment. Notably, patients with the highest RoPE scores (9–10) paradoxically have very low observed stroke recurrence rates (~2% at 2 years) on medical therapy, despite a high probability that the PFO caused the index stroke [24]. Thus, a high RoPE score alone should be interpreted with caution when deciding on closure. RoPE and PASCAL serve as general guides to risk stratification rather than definitive criteria.

### 6.3. Does PASCAL or PFO “Type” Influence Anticoagulation Failure?

Direct evidence that specific PFO subtypes or PASCAL categories are associated with failure of anticoagulation (i.e., recurrent events despite therapeutic OAC) is limited, as very few trials have been powered to analyze this interaction. However, several lines of inference support the hypothesis that high-risk anatomy predisposes to residual risk despite anticoagulation:

1. High-risk anatomical features (large shunt, ASA, hypermobile septum, prominent Eustachian valve/Chiari network, and low-angle PFO) are associated with higher baseline risk of paradoxical embolism and stroke recurrence on medical therapy [12,13,14,25].
2. The greatest relative and absolute benefit of closure over medical therapy in RESPECT, REDUCE, and DEFENSE-PFO was observed in patients with these high-risk morphologies [6,7,26].
3. Observational data suggest that recurrence rates in medically managed PASCAL “probable” PFO-AS may approach 1–2% per year, despite antithrombotic therapy [1,11].

Mechanistically, it is intuitive that large, low-angle shunts with flow-directing structures such as Eustachian valves or Chiari networks create a more efficient conduit for paradoxical emboli. Even small thrombi, incompletely suppressed by anticoagulation, may be preferentially directed through these channels.

Thus, while hard data are still emerging, current evidence and pathophysiological reasoning strongly suggest that patients in the PASCAL “probable” category with high-risk PFO anatomy are precisely those in whom anticoagulation is most likely to fail and closure is most likely to be superior, although this hypothesis has yet to be tested in a prospective trial.

## 7. Clinical Implications and Guideline Perspectives

The 2021 AHA/ASA guideline for secondary stroke prevention recommends that in patients aged 18–60 years with a non-lacunar ischemic stroke of undetermined cause and PFO with high-risk anatomic features, PFO closure is reasonable after a multidisciplinary discussion and shared decision-making (Class IIa, Level of Evidence B-R) [27]. For patients without high-risk features or with competing mechanisms of stroke, medical therapy (antiplatelet or, in selected situations, anticoagulation) is preferred.

European and other international guidelines are broadly concordant, emphasizing high-risk PFO anatomy and high RoPE scores as criteria supporting closure and acknowledging that the incremental benefit of anticoagulation over antiplatelets remains uncertain [11,25].

Anticoagulation as a long-term substitute for closure in high-risk, PASCAL-probable patients appears mechanistically and empirically suboptimal: residual stroke risk is non-trivial, and bleeding complications may accumulate over decades of therapy.

### 7.1. Limitations of Clinical Trials

Despite the supporting evidence described above, it is important to recognize the limitations of these trials and analyses. The absolute risk reductions observed with closure were small in magnitude (on the order of only 1% per year or less) [4,5,6], because stroke recurrence rates were low in both the closure and medical therapy groups. Some trials were not originally powered to detect even these modest differences and only achieved statistical significance with extended follow-up or pooled analyses [21,28,29]. No heterogeneity of closure effect was seen within the 18–60 age range of trials, but DEFENSE-PFO (which allowed older patients) still showed benefit, hinting that older patients might benefit if they have high-risk anatomy. Nevertheless, decision-making in an older patient with stroke and a PFO should consider competing stroke mechanisms (e.g., atrial fibrillation or small vessel disease that become more common with age) [7,29]. The positive PFO closure trials were open-label and enrolled predominantly young patients with few vascular risk factors, so their results may not generalize to older or more comorbid populations [4,5,6,27].

Moreover, unmeasured confounding could have influenced outcomes—for example, a notable proportion of patients in both arms were using estrogen–progestin therapy. In the CLOSE trial 41.6% vs. 39.8% and in the RESPECT trial 28.2% vs. 33.2% were using oral contraceptive pills between closure vs. medical arm, respectively. Hormonal contraceptives are a prothrombotic factor that was not accounted for in the analyses [4,5,30]. Likewise, in the CLOSE trial, the prevalence of migraines was 28.2% vs. 33.2%, and in the RESPECT trial it was 39.1% vs. 38.7%, between closure vs. medical arm, respectively. Migraine, like hormonal contraceptive use, was not stratified for outcome analysis-underlying unmeasured confounders [4,5].

Finally, atrial cardiopathy—patients with covert atrial fibrillation or significant left atrial enlargement were generally excluded or underrepresented in PFO trials. For patients with PFO and atrial cardiopathy, the stroke might not be PFO-mediated, and those patients might require anticoagulation regardless of PFO. Thus, closure’s benefit would be less clear in that scenario.

### 7.2. Limitations of RoPE and PASCAL Classification

Additionally, as discussed, the RoPE score and PASCAL classification tools have not been prospectively validated for guiding treatment; indeed, patients with very high RoPE scores (9–10) can have low 2-year recurrence rates (~2%) on medical therapy, even though such patients have the highest probability of PFO-AS [10,29,31]. PASCAL is a helpful framework but remains to be prospectively validated; thus, it should be used as a guide alongside clinical judgment rather than as a strict algorithm. These considerations urge caution: PFO closure should be offered primarily to those patients who closely resemble the trial populations (i.e., younger, cryptogenic stroke, and high-risk PFO anatomy), and even then the decision must weigh the modest absolute benefit against the potential risks [27,32].

In practice, the choice between transcatheter PFO closure and long-term anticoagulation should be individualized. Current guidelines advocate for shared decision-making in this context [27,32]. Factors such as a patient’s bleeding risk, values and preferences, and access to experienced interventional cardiology centers should be considered. PFO closure entails an invasive procedure with upfront risks (including a roughly 3–5% chance of device-related atrial fibrillation in trials) [4,5,6] and the costs of the device and hospitalization [33]. Conversely, indefinite anticoagulation carries cumulative risks of major bleeding and can impact quality of life (e.g., need for ongoing medication adherence and, for warfarin, regular INR monitoring) [34]. Some patients may strongly prefer a one-time procedure to avoid lifelong anticoagulation, while others may be averse to procedural intervention and accept the stroke and bleeding risks of medical therapy [32]. Therefore, the “best” strategy will vary by individual. A personalized, patient-centered approach—weighing the small absolute benefit of closure against its risks—is essential in this decision-making process.

## 8. Future Directions

Based on the current evidence and mechanistic understanding, several avenues for future research emerge.

### 8.1. Direct Comparison of DOACs vs. PFO Closure in PASCAL-Probable Patients

To date, no randomized trial has directly compared modern DOACs against PFO closure in a population enriched for PASCAL-probable PFO-AS (young, high RoPE, and high-risk anatomy). The anticoagulation data are largely extrapolated from warfarin-based regimens and from ESUS populations where PFO can be incidental.

A trial that randomizes such patients to DOACs vs. PFO closure plus antiplatelet therapy with stratification by PASCAL class and high-risk anatomical features would directly test whether any medical regimen can match closure in this high-risk group.

### 8.2. Refined Phenotyping of Atrial Cardiopathy in PFO-AS

Emerging evidence suggests that atrial cardiopathy may coexist with PFO, particularly in older patients or those with vascular risk factors, and may contribute to thromboembolism independently of paradoxical embolism [9,20].

Prospective studies incorporating advanced echocardiography (left atrial strain, volume, and stiffness), cardiac MRI, biomarkers (NT-proBNP, high-sensitivity troponin, and prothrombotic markers), and prolonged rhythm monitoring could identify subgroups in whom combined strategies (PFO closure plus anticoagulation) are warranted and those for whom closure alone is sufficient.

### 8.3. Mechanistic Imaging of Venous and in Situ Thrombus

Better understanding of where thrombi originate in PFO-AS is needed. Research combining systematic venous Doppler and pelvic MR venography for occult DVT, high-resolution imaging of PFO tunnels for in situ thrombus, and serial D-dimer and other coagulation markers could clarify the relative contributions of venous vs. in situ vs. atrial thrombus and inform which patients might still benefit from anticoagulation after closure.

### 8.4. Post-Closure Antithrombotic Optimization

Post-closure antithrombotic regimens vary widely (short-term dual antiplatelet therapy vs. single antiplatelet vs. short-term anticoagulation). Trials focusing on high-risk subsets (e.g., device-related thrombus, strong thrombophilia, and atrial cardiopathy) could optimize these regimens and minimize both stroke and bleeding risk.

### 8.5. Device Design and Procedure-Related Arrhythmias

A small but real increase in new-onset atrial fibrillation is observed after PFO closure, although most episodes are early and transient [4,6,7]. Future studies should define which devices and techniques minimize atrial fibrillation risk and device-related thrombosis and determine whether short-term prophylactic anticoagulation or tailored monitoring in high-risk patients improves outcomes.

### 8.6. Decision Tools Integrating PASCAL, Bleeding Risk, and Patient Preferences

Finally, development and validation of decision support tools that integrate PASCAL category, detailed anatomy, individual bleeding risk (HAS-BLED or similar), life expectancy and comorbidities, and patient values and preferences could guide personalized therapy—particularly in gray-zone scenarios where both closure and anticoagulation are plausible options.

## 9. Conclusions

PFO-AS lies at the intersection of anatomy, thrombosis, and atrial biology. Anticoagulation reduces but does not eliminate venous thrombus formation and leaves the anatomical conduit (PFO) intact, allowing paradoxical embolism to occur. High-risk anatomical PFO features favor persistent embolic risk despite anticoagulation. Randomized trials consistently show superiority of closure in selected patients—particularly those with large shunts, ASA, or high PASCAL “probable” classification in the trial populations studied. The concept of anticoagulation “failure” in PFO-AS should prompt clinicians to reassess both the mechanism (PFO-mediated vs. atrial or arterial) and the anatomy (high-risk vs. low-risk PFO), rather than simply intensifying or switching anticoagulant agents. It is acknowledged that the interpretation of “anticoagulation failure” in this context is based on synthesis of available evidence and has not yet been confirmed by direct comparison trials. In PASCAL-probable patients with high-risk anatomy, the weight of evidence and pathophysiology strongly supports closure as the preferred strategy. Anticoagulation should be reserved for established indications (atrial fibrillation, thrombosis, and thrombophilia) and used as an adjunct rather than a substitute when the PFO conduit is clearly causal. Future research must directly compare DOACs with closure in high-risk populations, refine our understanding of atrial cardiopathy in PFO-AS, and develop integrated decision tools to further individualize therapy.

## Figures and Tables

**Table 1:** Summary of key randomized trials of PFO closure vs. medical therapy for secondary stroke prevention.

Trial	N (Closure vs. Medical)	Population (Key Inclusion)	Medical Therapy Arm	Follow-Up (Median)	Recurrent Stroke Rate (Closure vs. Medical) and Effect Size	Atrial Fibrillation (Closure vs. Medical)	Notable Limitations
CLOSE [5]	238 vs. 235	High-risk PFO (ASA or large shunt; 16–60 years old)	Antiplatelet only	~5.3 years	0 vs. 14 strokes (0% vs. 6.0%); HR~0.03 (95% CI 0–0.26)	4.6% vs. 0.9% (peri-procedural AF in closure group)	Open-label; multiple device types used; warfarin was primary anticoagulant; closure vs. OAC not directly powered (small anticoagulation arm)
RESPECT [4]	499 vs. 481	Cryptogenic stroke with PFO (18–60 years old)	Antiplatelet or OAC (MD choice)	5.9 years	18 vs. 28 strokes (3.6% vs. 5.8%); HR 0.55 (95% CI 0.31–0.999), *p* = 0.046. Stroke of unknown cause: HR 0.38 (0.18–0.79).	~0.4% vs. ~0.2% per year (no significant difference)	Open-label; higher dropout in medical arm; benefit became significant only with extended (post-hoc) follow-up; device group had slightly more VTE events (possibly due to less warfarin use)
REDUCE [6]	441 vs. 223	Cryptogenic stroke with PFO; 81% had large or moderate shunt (18–59 years old)	Antiplatelet only	3.2 years	6 vs. 12 strokes (1.4% vs. 5.4%); HR 0.23 (95% CI 0.09–0.62), *p* = 0.002. New brain infarcts (clinical or silent): 4.7% vs. 10.7%, *p* = 0.02.	6.6% vs. 0.4% (mostly peri-procedural in closure arm)	Open-label; composite endpoint included MRI-detected silent infarcts (potential ascertainment bias); AF monitoring largely ceased after 30 days, possibly underestimating late AF in either group
DEFENSE-PFO [7]	60 vs. 60	High-risk PFO (ASA, large shunt or hypermobile septum; mean age 51.8 years)	Antiplatelet or warfarin	2.8 years	0 vs. 5 strokes (0% vs. 8.3%, *p* = 0.023); primary composite (stroke/vascular death/major bleed): 0 vs. 6 events (12.9%)	3.3% vs. 0% (2 patients with new-onset AF post-closure, 0 in medical arm)	Very small sample (stopped early at N = 120); wide confidence intervals; open-label design; conducted in a single country (South Korea) with limited generalizability; comparator included mixed medical therapies

## Data Availability

Data supporting the conclusions are derived from previously published literature cited in the manuscript; further details can be provided by the author upon request.

## References

[1] Kent D.M., Wang A.Y. (2025). Patent foramen ovale and stroke: A review. JAMA.

[2] Horton S.C., Bunch T.J. (2004). Patent foramen ovale and stroke. Mayo Clin. Proc..

[3] Shah A.H., Horlick E.M., Kass M., Carroll J.D., Krasuski R.A. (2024). The pathophysiology of patent foramen ovale and its related complications. Am. Heart J..

[4] Saver J.L., Carroll J.D., Thaler D.E., Smalling R.W., MacDonald L.A., Marks D.S., Tirschwell D.L. (2017). Long-term outcomes of patent foramen ovale closure or medical therapy after stroke. N. Engl. J. Med..

[5] Mas J.-L., Derumeaux G., Guillon B., Massardier E., Hosseini H., Mechtouff L., Arquizan C., Béjot Y., Vuillier F., Detante O. (2017). Patent foramen ovale closure or anticoagulation vs. antiplatelets after stroke. N. Engl. J. Med..

[6] Søndergaard L., Kasner S.E., Rhodes J.F., Andersen G., Iversen H.K., Nielsen-Kudsk J.E., Settergren M., Sjöstrand C., Roine R.O., Hildick-Smith D. (2017). Patent foramen ovale closure or antiplatelet therapy for cryptogenic stroke. N. Engl. J. Med..

[7] Lee P.H., Song J.-K., Kim J.S., Heo R., Lee S., Kim D.-H., Song J.-M., Kang D.-H., Kwon S.U., Kang D.-W. (2018). Cryptogenic stroke and high-risk patent foramen ovale: The DEFENSE-PFO trial. J. Am. Coll. Cardiol..

[8] Giacoppo D., Caronna N., Frangieh A.H., Michel J., Andò G., Tarantini G., Kasel A.M., Capodanno D., Byrne R.A. (2018). Long-term effectiveness and safety of transcatheter closure of patent foramen ovale compared with antithrombotic therapy alone: A meta-analysis of six randomised clinical trials and 3,560 patients with reconstructed time-to-event data. EuroIntervention.

[9] Li W., Zhang J., Zhang Y., Shentu W., Yan S., Chen Q., Qiao S., Kong Q. (2025). Clinical research progress on pathogenesis and treatment of Patent Foramen Ovale-associated stroke. Front. Neurol..

[10] Kent D.M., Saver J.L., Kasner S.E., Nelson J., Wang A.Y., Bannuru R.R., Carroll J.D., Chatellier G., Derumeaux G., Furlan A.J. (2023). Evaluating Therapies to Prevent Future Stroke in Patients with Patent Foramen Ovale-Related Strokes—The SCOPE Study.

[11] Sposato L.A., Albin C.S., Elkind M.S., Kamel H., Saver J.L. (2024). Patent foramen ovale management for secondary stroke prevention: State-of-the-art appraisal of current evidence. Stroke.

[12] Nakayama R., Takaya Y., Akagi T., Miki T., Nakagawa K., Toh N., Ito H. (2021). Low-angle patent foramen ovale (PFO): High-risk PFO morphology associated with paradoxical embolism. CASE.

[13] Zhang H., Liu W., Ma J., Liu H., Li L., Zhou J., Wang S., Li S., Wang W., Wang Y. (2022). Pitfalls of using imaging technique in the presence of eustachian valve or chiari network: Effects on right-to-left shunt and related influencing factors. Diagnostics.

[14] Onorato E.M. (2021). Large eustachian valve fostering paradoxical thromboembolism: Passive bystander or serial partner in crime?. World J. Cardiol..

[15] Martínez-Quintana E., Rodríguez-González F. (2015). Chiari network and paradoxical embolism. Rev. Esp. Cardiol. (Engl. Ed.).

[16] Manerikar A., Malaisrie S.C. (2022). Chiari network and patent foramen ovale associated with stroke. JTCVS Tech..

[17] Kent D.M., Dahabreh I.J., Ruthazer R., Furlan A.J., Weimar C., Serena J., Meier B., Mattle H.P., Di Angelantonio E., Paciaroni M. (2015). Anticoagulant vs. antiplatelet therapy in patients with cryptogenic stroke and patent foramen ovale: An individual participant data meta-analysis. Eur. Heart J..

[18] Kasner S.E., Swaminathan B., Lavados P., Sharma M., Muir K., Veltkamp R., Ameriso S.F., Endres M., Lutsep H., Messé S.R. (2018). Rivaroxaban or aspirin for patent foramen ovale and embolic stroke of undetermined source: A prespecified subgroup analysis from the NAVIGATE ESUS trial. Lancet Neurol..

[19] Mir H., Siemieniuk R.A.C., Ge L.C., Foroutan F., Fralick M., Syed T., Lopes L.C., Kuijpers T., Mas J.-L., Vandvik P.O. (2018). Patent foramen ovale closure, antiplatelet therapy or anticoagulation in patients with patent foramen ovale and cryptogenic stroke: A systematic review and network meta-analysis incorporating complementary external evidence. BMJ Open.

[20] Pirera E., D’Anna L., Di Raimondo D., Tuttolomondo A. (2024). Direct oral anticoagulants versus aspirin for stroke prevention after embolic stroke of undetermined source: An updated Meta-Analysis of randomized controlled trials. J. Clin. Med..

[21] Meier B., Kalesan B., Mattle H.P., Khattab A.A., Hildick-Smith D., Dudek D., Andersen G., Ibrahim R., Schuler G., Walton A.S. (2013). Percutaneous closure of patent foramen ovale in cryptogenic embolism. N. Engl. J. Med..

[22] Hart R.G., Sharma M., Mundl H., Kasner S.E., Bangdiwala S.I., Berkowitz S.D., Swaminathan B., Lavados P., Wang Y., Wang Y. (2018). Rivaroxaban for stroke prevention after embolic stroke of undetermined source. N. Engl. J. Med..

[23] Wiktor D.M., Carroll J.D. (2018). The case for selective patent foramen ovale closure after cryptogenic stroke. Circ. Cardiovasc. Interv..

[24] Ioannidis S.G., Mitsias P.D. (2020). Patent foramen ovale in cryptogenic ischemic stroke: Direct cause, risk factor, or incidental finding?. Front. Neurol..

[25] Forno D., Asteggiano R., Parrini I. (2025). Patent foramen ovale in patients with cryptogenic stroke: To close or not to close?. Eur. Soc. Cardiol..

[26] Carroll J.D., Saver J.L., Thaler D.E., Smalling R.W., Berry S., MacDonald L.A., Marks D.S., Tirschwell D.L. (2013). Closure of patent foramen ovale versus medical therapy after cryptogenic stroke. N. Engl. J. Med..

[27] Kleindorfer D.O., Towfighi A., Chaturvedi S., Cockroft K.M., Gutierrez J., Lombardi-Hill D., Kamel H., Kernan W.N., Kittner S.J., Leira E.C. (2021). 2021 guideline for the prevention of stroke in patients with stroke and transient ischemic attack: A guideline from the American Heart Association/American Stroke Association. Stroke.

[28] Furlan A.J., Reisman M., Massaro J., Mauri L., Adams H., Albers G.W., Felberg R., Herrmann H., Kar S., Landzberg M. (2012). Closure or medical therapy for cryptogenic stroke with patent foramen ovale. N. Engl. J. Med..

[29] Kent D.M., Saver J.L., Kasner S.E., Nelson J., Carroll J.D., Chatellier G., Derumeaux G., Furlan A.J., Herrmann H.C., Jüni P. (2021). Heterogeneity of treatment effects in an analysis of pooled individual patient data from randomized trials of device closure of patent foramen ovale after stroke. JAMA.

[30] Lidegaard Ø., Løkkegaard E., Svendsen A.L., Agger C. (2009). Hormonal contraception and risk of venous thromboembolism: National follow-up study. BMJ.

[31] Kent D.M., Ruthazer R., Weimar C., Mas J.-L., Serena J., Homma S., Di Angelantonio E., Di Tullio M.R., Lutz J.S., Elkind M.S. (2013). An index to identify stroke-related vs. incidental patent foramen ovale in cryptogenic stroke. Neurology.

[32] Caso V., Turc G., Abdul-Rahim A.H., Castro P., Hussain S., Lal A., Mattle H., Korompoki E., Søndergaard L., Toni D. (2024). European Stroke Organisation (ESO) Guidelines on the diagnosis and management of patent foramen ovale (PFO) after stroke. Eur. Stroke J..

[33] Leppert M.H., Poisson S.N., Carroll J.D., Thaler D.E., Kim C.H., Orjuela K.D., Ho P.M., Burke J.F., Campbell J.D. (2018). Cost-effectiveness of patent foramen ovale closure versus medical therapy for secondary stroke prevention. Stroke.

[34] Parks A.L., Slager S.L., Cizik A.M., Fang M.C., Supiano M.A., Katz P.P., Witt D.M. (2025). The impact of anticoagulant-related bleeding on quality of life: Development of a novel measure based on perspectives from older adults. PLoS ONE.

